# Distorting the metric fabric of the cognitive map

**DOI:** 10.1016/j.tics.2015.04.001

**Published:** 2015-06

**Authors:** Kate J. Jeffery

**Affiliations:** Institute of Behavioural Neuroscience, Department of Experimental Psychology, University College London, 26 Bedford Way, London WC1H 0AP, UK

**Keywords:** spatial perception, grid cells, place cells, boundaries, environment geometry, entorhinal cortex

## Abstract

Grid cells are neurons whose regularly spaced firing fields form apparently symmetric arrays, or grids, that are thought to collectively provide an environment-independent metric framework for the brain's cognitive map of space. However, two recent studies show that grids are naturally distorted, revealing greater local environment-specific effects than previously recognized.

## A metric framework for the cognitive map?

**Figure 1:**
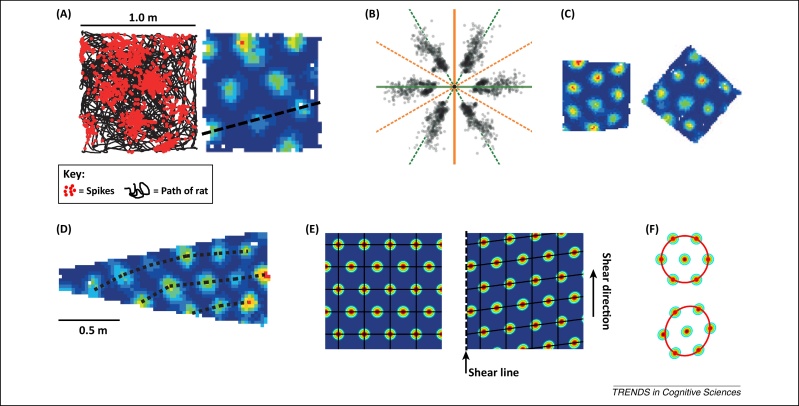
Grid-cell grids are less symmetric and universal than was thought. **(A)** Left: The raw data from a typical grid cell (adapted and reprinted by permission from Macmillan Publishers Ltd: *Nature*[2], copyright 2015), recorded as a rat foraged for several minutes in a square box, showing clusters of action potentials (red circles) arranged in regularly spaced rows. Right: A rate map, which is a heat plot of the spatial firing of the same cell. The black broken line highlights a row of firing fields. **(B)** Modified figure from [3] (adapted and reprinted by permission from Macmillan Publishers Ltd: *Nature*[3], copyright 2015) showing that the distribution of the orientation of grid-field rows was clustered rather than random. The angular position of each gray circle represents the direction of one of the cell's major axes (six for each cell) and the radial position the inter-field distance (‘scale’). The unbroken lines indicate the two major axes of the square recording environment. Although the cells all have one of their major axis-clusters near one of the lines, the alignment of the clusters is not perfect – the array of data points is rotated away from the axis by about +/− 8 degrees. **(C)** Rotation of a square box in [2] reveals a strong influence of the box walls on the orientation of the grid pattern. **(D)** Rate map of the same cell as in **(A)**, this time recorded in a trapezoidal enclosure, showing how the rows of firing fields are distorted by the angled walls. **(E)** The shearing hypothesis of [3]. The left plot shows an idealized symmetric grid-cell array: black lines provide a reference grid. The right plot shows the effect of a shearing transformation, anchored at the left-hand edge (the shear line – broken black line), at which points do not move, and with the remaining points moving in the shear direction by an amount proportional to their distance from the shear line. **(F)** Such a shear transformation, applied to a hexagonal unit within a grid, converts the circular array of firing fields into a tilted ellipse.

Making one's way in the spatial world requires an internal representation of space, often called a cognitive map, which requires the processing of distances and directions. In service of this aim, place cells in the hippocampus encode spatial locations whereas grid cells in entorhinal cortex may encode the twin metrics of distance and direction. The entorhinal metric hypothesis arose because grid cells show patches of activity, or firing fields, that are spread in regularly spaced rows across the surface of an environment [1] (Figure 1A) in a grid-like array (hence their name), suggesting that the cells might operate like the grid reference on a map to allow spatial computations, for example by place cells.

Grids appear highly regular and symmetric when an animal enters a new environment and were thus thought to be governed by factors independent of the local environment: an orientation signal set by distant directional cues, together with internal network factors that determine the scale (distance between firing fields). Neither orientation nor scale should thus be affected by the nearby boundaries. However, two studies recently published in the same issue of *Nature*, by Krupic *et al.* [2] and Stensola *et al.* [3], challenge this view. Their findings suggest that boundaries are more important than was previously thought, such that the stable end-point of a grid is not necessarily hexagonally symmetric. These observations raise questions about our original assumption that grid cells form a universal environment-independent spatial metric, and also provide clues as to how the grids anchor to the local environment.

## A distorted metric

Both studies began with detailed measurements of grid cell grids in a symmetric, square environment. The first surprising observation was that there was a highly non-random clustering of grid orientations: for a given cell, the grid would be oriented such that a row of fields would tend to follow the alignment of one of the walls (Figure 1B). Krupic *et al.* confirmed the strong influence of boundaries by rotating the box and observing that grids rotated too (Figure 1C). These observations conflict with the previous assumption that grid orientation is established by distal environmental cues: clearly, this is not necessarily the case.

The second surprising observation about grid orientation, made by both studies, is that the field rows did not exactly align with the boundaries; in fact, they were rotated off-axis by a few degrees (8.8 in [2] and 7.4 in [3]; Figure 1B). Third, even when environments were familiar, the grids did not show the expected symmetry. This was revealed in two ways. Krupic *et al.* recorded grids in a trapezoid-shaped enclosure and found that the grid pattern was distorted (Figure 1D), with greater spacing between fields and variation from one end of the box to the other in how the fields were aligned. Stensola *et al*., continuing with the square-box studies, found that after rats became familiar with the environment the grids became compressed in one direction. Interestingly, both the off-axis tilting and the compression developed with experience. In the largest square (2.2 m on a side) this occurred in complex ways that suggested greatest influence from nearby boundaries. The authors modelled this distortion and showed that it is mathematically well described by a shearing transformation applied to a regular grid (Figure 1E), as if the enclosure walls were somehow dragging or pushing on the grids, but – importantly – in a non-uniform way (or else, since the box is symmetrical, the grid would just stretch or squash equally in all directions). Such a shearing transformation could explain both the rotation of the grid rows away from the box walls, as well as the squashing of the grid pattern (Figure 1F).

The necessary conclusion from both of these studies is that the grid pattern in a stable, familiar environment is not fully pre-ordained; rather, grids settle into a steady-state pattern that is the outcome of an interaction between the pre-configured factors of orientation and scale (which the rat brings in with it, as it were) and the local environment boundaries.

## Causes and consequences of grid distortion

These findings raise important questions. Notably, what causes these effects, and what are the implications for how grid cells might encode space?

The causes of environmental grid distortion can currently only be guessed at, but clearly they involve the environment boundaries. An earlier study in which a familiar environment was deformed found that grids became partially deformed as well [4], suggesting that the grids had become ‘attached’ to the boundaries with experience. The latest studies suggest that the influence of the boundaries is more than simply stabilization. Because the stable end-point of the grid cell is evidently a distorted grid even in a non-deformed enclosure, the implication is that boundaries are always exerting some kind of deforming influence, even if the environment is stable; that is, they are always either pushing or pulling on grids. One possible mediator of this influence is border cells, found in areas closely related to those of grid cells [5]. These cells fire along boundaries and might excite or inhibit grid cells.

All these border-related influences are symmetric, so what breaks the symmetry to allow distortion of the grid in only one direction? Stensola *et al.* suggested that this occurs as a result of one or more fixed points; places in the environment where the grid is anchored and thus resists being pushed or pulled. With a combination of fixed attachment points, together with a sustained force from the environment boundaries, grids could thus become anisotropically deformed even in stable, symmetric environments.

What does grid distortion mean, functionally speaking? Stensola *et al.* noted that a 7.5 degree tilt of the major axis maximally reduces the symmetry of the overall arrangement in a square enclosure; however it is not yet clear if this is relevant, or useful. It seems more likely that the effect is an accidental outcome of two functionally adaptive processes: attachment to fixed environmental points plus guidance from boundaries, both of which could be used to help position and stabilize the grid as the animal moves around. However, might the resulting grid distortions cause distortions in navigational calculations? If grids are used in navigation then the answer is probably ‘yes, but only slightly’. However, it could be that grids are used for something else; what that might be, we do not yet know.
